# Further Examination of the Pulsed- and Steady-Pedestal Paradigms under Hypothetical Parvocellular- and Magnocellular-Biased Conditions

**DOI:** 10.3390/vision8020028

**Published:** 2024-04-30

**Authors:** Jaeseon Song, Bruno G. Breitmeyer, James M. Brown

**Affiliations:** 1 Department of Psychology, University of Georgia, Athens, GA 30602, USA; jmbrown@uga.edu; 2 Department of Psychology, University of Houston, Houston, TX 77204, USA; brunob@uh.edu

**Keywords:** contrast discrimination, magnocellular, parvocellular

## Abstract

The pulsed- and steady-pedestal paradigms were designed to track increment thresholds (Δ*C*) as a function of pedestal contrast (C) for the parvocellular (P) and magnocellular (M) systems, respectively. These paradigms produce contrasting results: linear relationships between Δ*C* and *C* are observed in the pulsed-pedestal paradigm, indicative of the P system’s processing, while the steady-pedestal paradigm reveals nonlinear functions, characteristic of the M system’s response. However, we recently found the P model fits better than the M model for both paradigms, using Gabor stimuli biased towards the M or P systems based on their sensitivity to color and spatial frequency. Here, we used two-square pedestals under green vs. red light in the lower-left vs. upper-right visual fields to bias processing towards the M vs. P system, respectively. Based on our previous findings, we predicted the following: (1) steeper Δ*C* vs. *C* functions with the pulsed than the steady pedestal due to different task demands; (2) lower Δ*C*s in the upper-right vs. lower-left quadrant due to its bias towards P-system processing there; (3) no effect of color, since both paradigms track the P-system; and, most importantly (4) contrast gain should not be higher for the steady than for the pulsed pedestal. In general, our predictions were confirmed, replicating our previous findings and providing further evidence questioning the general validity of using the pulsed- and steady-pedestal paradigms to differentiate the P and M systems.

## 1. Introduction

The parvocellular (P) and magnocellular (M) pathways in the visual system exhibit distinct characteristics in terms of spatiotemporal responses [1,2,3,4,5,6,7,8], contrast gain [1,2,3,9,10], and color [7,11,12,13,14,15] processing. This differentiation has allowed for psychophysical studies aimed at isolating and understanding the specific functions of the P and M systems. Through these studies, it has been established that the M pathway is particularly tuned to detect motion and rapid changes, exhibiting a preference for low spatial and high temporal frequencies, which is consistent with neurophysiological research on macaques (see Figure 2 in [16]). Moreover, the M pathway is characterized by a higher contrast gain compared to the P system [17,18]. Research has also uncovered that exposure to diffuse red light can significantly diminish the activity of the M system throughout the visual pathway, from the retina to the cortex [19,20,21].

**Figure 1:**
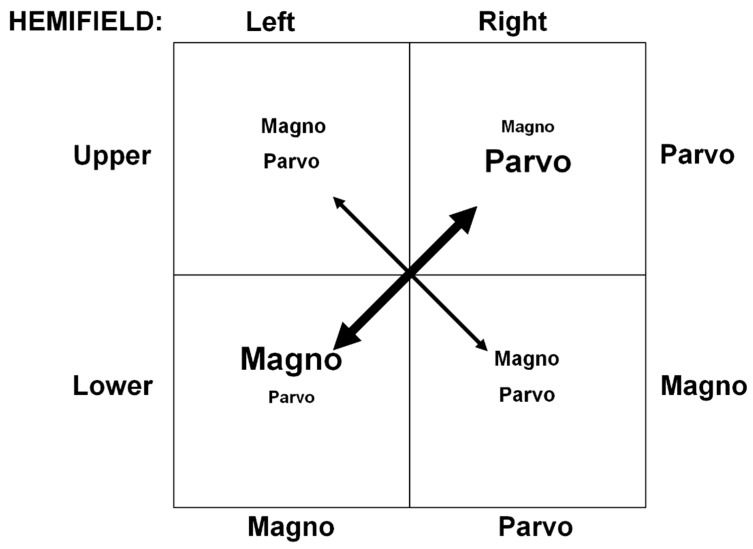
Visual hemifield (upper vs. lower and left vs. right) differences in preferential M- and P-activation patterns. The resulting strongest preferences, depicted by the bold double arrow, of the M and the P systems, are found, respectively, in the lower-left and upper-right VFs. Progressively larger activation biases are given by entries with progressively larger print.

In addition, based on theoretical and empirical research, it is possible that there may be a connection between upper–lower and left–right visual-field (VF) asymmetries regarding the distinct contributions of the P and M systems to visual processing. We base our reasoning on a combination of theoretical and empirical reviews provided by Kosslyn and collaborators [22,23] on one hand and by Previc [24,25] on the other. The former researchers proposed firstly that there are hemispheric and corresponding visual-field differences between the processing of categorical and coordinate spatial relationships, with the left hemisphere and right visual field (RVF) specializing in categorical judgments and the right hemisphere and left visual field (LVF) specializing in coordinate judgments. Secondly, they and others [26,27,28] also proposed that high-level visual areas in the left and right cortical hemispheres are characterized by neural systems responding preferably to higher and lower spatial frequencies, respectively. Reasonably taking these two systems to correspond to the parvo (P)- and magnocellular (M) systems, respectively, we propose that RVF and LVF spatial stimuli were processed preferentially by the P and M systems, respectively. Additionally, based on extensive empirical findings, Previc argued that the lower visual field (LVF) and the upper visual field (UVF) are biased toward M-system and P-system processing, respectively. Based on this line of reasoning, Kosslyn et al.’s and Previc’s proposals support our hypothesis, as depicted in Figure 1, that the upper-right and lower-left quadrants of the visual field are maximally biased toward P-system and M-system processing, respectively. Findings consistent with this hypothetical scheme have been reported in Niebauer and Christman’s study [29] on categorical and coordinate spatial relations and Peyrin et al.’s event-related fMRI [26,28] and psychophysical [27] studies on scene recognition.

Pokorny and Smith [10] developed a psychophysical technique to differentiate P and M systems based on their characteristic contrast gain and temporal-integration signatures. This technique has been widely used to explore a number of aspects of both normal vision, e.g., [1,2,30,31], and abnormal vison, e.g., [3,32,33,34,35,36], as well as other neural and cognitive disorders, e.g., [37,38,39]. The steady-pedestal and pulsed-pedestal paradigms, designed to examine contrast detection and discrimination, are believed to target the M and P systems, respectively. In the steady-pedestal paradigm, a constant visual backdrop, or “pedestal,” made up of, for example, four squares, is presented. One of these squares briefly changes luminance to serve as the test stimulus, requiring observers to identify which square changed. This method is thought to favor the M pathway, known for its sensitivity to brief luminance changes. In contrast, the pulsed-pedestal paradigm introduces the pedestal and the target stimulus simultaneously but briefly. This method is hypothesized to primarily engage the P pathway, because the abrupt onset presumably saturates the transient response of the relatively more sensitive M system, leaving the slower, more sustained responding P system to detect the test stimulus.

Recently, Hugrass et al. [30] used the two paradigms to assess the impact of red backgrounds on M and P processing. Surprisingly, their findings showed no significant differences between results obtained with red and green backgrounds, leading them to conclude that the use of a red background did not effectively suppress M-system activity. Given previous studies that had reported evidence of such suppression, we were intrigued by why they found no evidence for the suppressive effect of red light. In our recent study [40], we applied Pokorny and Smith’s pedestal paradigms and used colored Gabor stimuli, presented on the same colored background, as pedestals to examine the effects of red vs. green backgrounds on the contrast-increment threshold (Δ*C*) as a function of the Gabor-pedestal contrast (*C*). Like Hugrass et al., we observed that the background color (red vs. green) failed to influence increment thresholds. However, the contrast gain values obtained from the Δ*C*s using the two paradigms challenged the hypothesis that the steady-pedestal paradigm predominantly tracks the M-system response. Instead, they suggested that both pulsed- and steady-pedestal paradigms primarily track the P-system response. Hence, the paradigms’ inabilities to distinguish between the M and P responses may explain the absence of evidence for the suppressive effect of red light obtained by Hugrass et al. and Song et al. [40].

However, despite the contrast gain values we found, another potential explanation for the failure to observe the suppressive effect of the red background in our previous study using Gabor pedestals might be linked to our deviation from the traditional approach established by Pokorny and Smith [10], where square pedestals were commonly used. The importance of pedestal shape is emphasized when considering receptive-field response characteristics. Only a subclass of M cells, the type-IV cells, are suppressed by uniform red light [21]. Hence, the red Gabor pedestals we used in our prior study [40] may not have been optimal in suppressing the type-IV M cells, since the red Gabor stimuli alternate between spatial increments of red light, leading to an increase in suppressive effects, and decrements of red light, leading to a decrease in suppressive effects. Pedestals consisting of spatially uniform increments of red light should therefore optimize the suppressive effects. We consequently decided to re-evaluate our previous assessment of the steady-pedestal paradigm’s and the pulsed-pedestal paradigm’s abilities to distinguish between the P and M systems. In the present study, we employed conventional square pedestals consisting of spatially uniform increments to examine the effectiveness of these paradigms as a function of the P and M systems’ contrast gains, VF asymmetries, and their respective response to equiluminant red and green colors. 

Pokorny and Smith [10] developed their steady- and pulsed-pedestal paradigms by utilizing the *Michaelis–Menten* equation, which describes the contrast-response functions (CRFs) of P and M cells [18]:

(1) $$R\left(C\right)=R_{0}+R_{max}C/\left(C_{sat}+C\right),$$

where *R* is the cell response, $R_{0}$ is the spontaneous activity, *R_max_* is the maximal (saturated) response, and *C_sat_* is the semisaturation contrast (the contrast at which the response is half of *R_max_*). *C_sat_* typically exhibits lower values for M cells, whereas *R*_0_ and *R_max_* can often be similar in both the P and M systems [41]. The initial slope of the CRF, represented as the percent contrast gain, (*R_max_/C_sat_*)/100, tends to be significantly higher in the M system compared to the P system, exemplified by the observation that the contrast gain in retinal M cells was approximately 10-times higher than in LGN P cells [19].

In addition, Pokorny and Smith [10] introduced an increment-threshold response criterion, denoted as *δ*, into the neural response function of Equation (1). This addition enabled them to establish a connection between their behavioral increment thresholds, ∆C, and the subsequent equation:

(2) $$∆C=\left(\delta /R_{max}\right){\left(C_{sat}+C\right)}^{2}/\left[C_{sat}-\left(\delta /R_{max}\right)\left(C_{sat}+C\right)\right].$$

To address the potential combination or summation of cells at stages beyond the retinal level, Pokorny and Smith [10]; see also [9] used a level-neutral adaptation of Equation (2), represented as

(3) $$\Delta C=K\left(10/R_{max}\right){\left(C_{sat}+C\right)}^{2}/\left[C_{sat}-\left(10/R_{max}\right)\left(C_{sat}+C\right)\right]$$

By setting *C_sat_* to 1.0, they derived the best-fitting estimates of the other two parameters: *K*, representing a scaling factor, and *R_max_*. In our study, we will likewise apply Equation (2) to obtain level-neutral estimates, not only for *R_max_* but also for *C_sat_* and *δ* as we did in our previous research [40]. The estimates that yield the best fit to our data will enable us to compute the percent contrast gain, expressed as (*R_max_*/*C_sat_*)/100.

**Figure 2:**
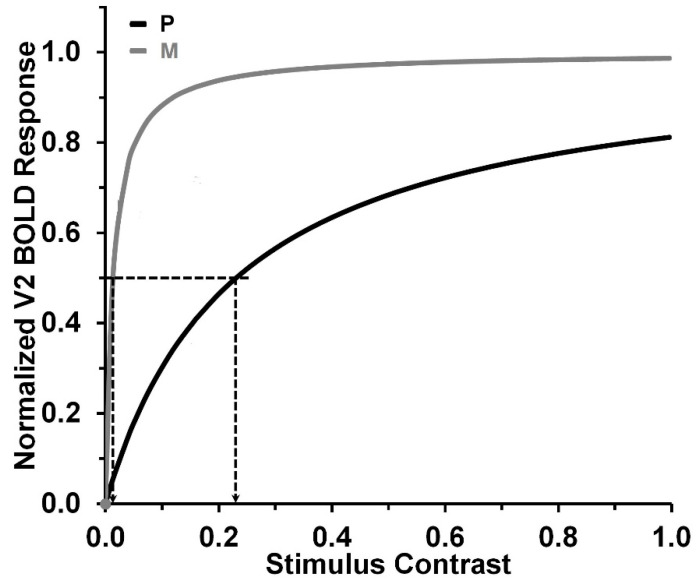
Best-fitting normalized CRF, modeled with the Michaelis–Menten formula, for BOLD responses obtained from human V2 P-innervated thin stripes and V2 M-innervated thick stripes. Dashed lines indicate *C_sat_* values. Adapted from Tootell and Nasr [50].

Given that higher post-retinal mechanisms likely contribute to psychophysical contrast detection and discrimination along the retino-geniculo-cortical pathways, we investigated whether contributions at higher levels might be involved in influencing psychophysical performance [40]. The rationale here is that the nonlinearity of the CRFs becomes more pronounced as we progress from the retina to the LGN [42,43,44,45,46], and further to the cortical regions [47,48,49]. In the retina, the P system is known for its nearly linear response as a function of stimulus contrast, but this characteristic changes as we progress along the visual pathway. As depicted in Figure 2, the CRFs in the extrastriate area V2 of the human visual cortex are noticeably nonlinear for both the P and M systems. The CRF curve of the M system in V2 rises sharply at low contrasts, as indicated by its low *C_sat_* value of 0.013. On the other hand, the CRF curve of the P system in V2, while rising more gradually, still exhibits a relatively steep slope with a *C_sat_* value of 0.231.

In general, the M system tends to exhibit significantly lower *C_sat_* values and higher contrast-gain values compared to the P system. This holds for the M system along the entire retino-geniculo-cortical pathway [18,50]. For example, contrast-gain values obtained from P-retinal ganglion cells are typically around 1 or lower, whereas the values obtained from M cells typically fall within the range of 1.5 to 4.5 [18]. The significance of this difference between the P and M systems, along with the overall trend of increasing nonlinearity (manifested as decreasing *C_sat_* values) in the CRFs along the retino-geniculo-cortical visual pathways, will be more apparent in the forthcoming presentation and discussion of our findings.

As noted above, our investigation centered around the variation of increment thresholds (Δ*C*) as a function of pedestal contrast (C) when presenting stimuli in two distinct VFs (see Figure 1): the lower-left VF, preferred by the M system, and the upper-right VF, favored by the P system. Although we also aimed to assess the suppression of the M system under red light compared to green light, we based our following hypotheses on our previous findings [40] by assuming that only the P system is involved, regardless of stimulus color:

1. The pulsed-pedestal paradigm would yield a steeper Δ*C* vs. *C* function in comparison to the steady-pedestal paradigm. This expectation arose from the premise that the pulsed-pedestal requires both detection and discrimination (i.e., a more difficult task), whereas the steady pedestal primarily focuses on discrimination (i.e., an easier task);
2. Our expectations regarding visual-field influences on Δ*C*s varied for the pulsed-pedestal and steady-pedestal paradigms, in part due to these task-demand differences. Overall, Δ*C*s were expected to be lower in the upper-right vs. lower-left VF due to the bias for P-system processing in the upper-right VF. Our expectations were less clear about Δ*C*s in the lower-left VF due our position that both paradigms target the P-system and our uncertainty about how the bias towards M-system processing there would interact with the task demands of the two paradigms. For the pulsed-pedestal paradigm, Δ*C*s were expected to be lower in the upper-right VF compared to the lower-left. This hypothesis was in line with both our stance and Pokorny and Smith’s [10] because both the upper-right VF and the pulsed-pedestal paradigm would be predominantly influenced by P-system processing. Our expectations diverge from Pokorny and Smith’s because of our position that the steady-pedestal paradigm also targets the P-system. According to Pokorny and Smith’s research, Δ*C*s should be lower in the lower-left than in the upper-right VF (i.e., opposite of the pulsed pedestal) reflecting the combined influence of the steady-pedestal paradigm targeting the M-system and the stronger influence of M-system processing in the lower-left VF. By comparison, we anticipated a conflict in the bias towards the M and P systems in response to stimuli presented in the M-biased lower-left VF under the P-biased steady-pedestal paradigm. This conflict might negate the pattern predicted by Pokorny and Smith or possibly even reverse the pattern of results.
3. The Δ*C*s under red and green light should be similar, as both paradigms predominantly engage the P-system. Therefore, any suppressive effect of red light on the M-system should not impact P-system mediated performance.
4. The M-system exhibits higher contrast-gain values than those for the P-system [18,50]. Nonetheless, the contrast-gain values for the steady-pedestal paradigm would not be higher than those for the pulsed-pedestal paradigm, as reported by Song et al. [40].

## 2. Materials and Methods

**Participants.** The participant group consisted of six individuals from the University of Georgia, including one faculty member, four graduate students, and one undergraduate student. One of the participants was female. To ensure that our sample size would be adequate for detecting the main effects of pedestal contrasts, we performed a power analysis using the “Bias and Uncertainty Corrected Sample Size” (BUCSS) R package (version 1.2.1) [51]. Unlike traditional methods, BUCSS calculates the required sample sizes for future studies based on observed t or F values and existing sample sizes, rather than relying on estimated effect sizes. From our previous study [40], we used the F values of the main effects of the pedestal paradigms (*F*(1, 6) = 73.97, *p* < 0.001) and pedestal contrasts (*F*(1.257, 7.542) = 105.71, *p* < 0.001). The power analyses indicated a minimum sample size of four and three, respectively, when assuming an alpha level of 0.05 and a desired statistical power level of 0.8. We chose six participants to take a conservative approach.

All participants were at least 18 years old at the time of the experiment and had normal or corrected-to-normal vision, as well as normal color vision (confirmed through pseudoisochromatic plates). The research adhered to the ethical guidelines for research involving human participants set by the University of Georgia Institutional Review Board and received their approval. 

**Instrumentation.** We used PsychoPy v.2021.2.3 [52] to create all test stimuli, which were displayed on a ViewSonic G90fB Graphics Series monitor. This monitor, set at an 85-Hz refresh rate and a resolution of 800 × 600, was calibrated using a Photo Research PR-650 Spectrophotometer. In the testing room, the monitor served as the sole light source. Participants viewed it binocularly from a distance of 70 cm, with their chin placed on a rest. 

**Stimuli.** The background luminance was consistently set at 5 cd/m^2^ for the duration of the experiment. For the red background, the CIE xy chromaticity coordinates were (x = 0.63, y = 0.33), and for the green background, they were (x = 0.30, y = 0.58). The test stimulus consisted of two squares. We tested two colors, physically equiluminant red and green, and six pedestal Michelson contrasts (*L*_max_ − *L*_min_/*L*_max_ + *L*_min_), 0.0, 0.08, 0.16, 0.32, 0.48, and 0.64, of the pedestal squares, where *L*_max_ was the variable luminance (cd/m^2^) of the pedestal and *L*_min_ was the fixed luminance of the background (5 cd/m^2^). The pedestal squares were 1.6° × 1.6° separated by 0.2° and presented in either the upper-right or lower-left VF at 3.6° eccentricity. The pedestal contrast represented an increase from the background’s 0.00 contrast level, making the pedestal invisible at its lowest contrast setting of 0.0 in both paradigms. The experiment consisted of 48 stimulus conditions (2 pedestal paradigms × 2 background colors × 2 quadrants of VF × 6 pedestal contrasts). For both pedestal paradigms, contrast increment thresholds, Δ*C*s, were computed relative to the Michelson contrast of the pedestal.

**Figure 3:**
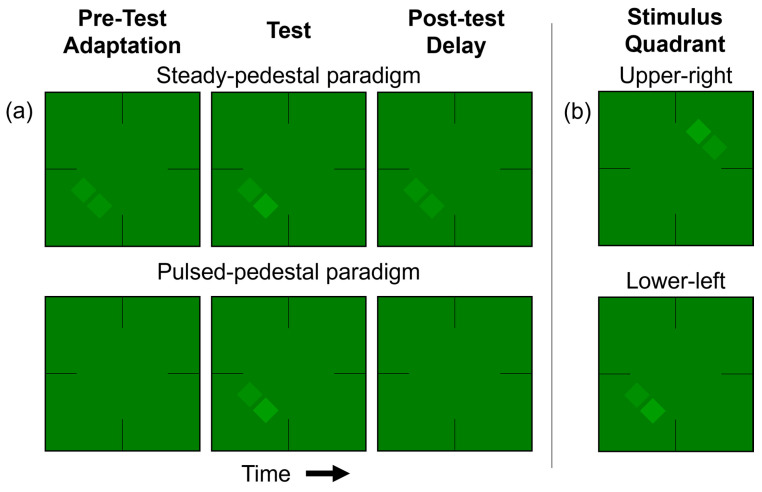
(**a**) The steady- and pulsed-pedestal paradigms for the lower-left VF condition. For the steady-pedestal paradigm (top row), a two-squares-array pedestal was presented continuously in the center of a constant surround. In each trial, one of the two squares was randomly selected to have an increased luminance, designating it as the test square, while the other remained unaltered, serving as the reference. During the testing interval, this luminance-increased test square was briefly displayed (35 ms). In the pulsed-pedestal paradigm (illustrated in the bottom row), participants initially adapted to the surrounding luminance. Then, in the test interval, both the test and reference squares were presented at the same time. For both paradigms, guides to aid fixation were consistently visible. The two-square array and its background were uniformly either red or green, both set to a physical equiluminance. (**b**) The test examples are of the upper-right VF (top) and the lower-left VF (bottom) conditions. The increment is on right in the lower-left VF example and on left in the upper-right example.

**Procedure (Experiment)**. Similar to our previous study [40], under both paradigms, a red or green two-square array was presented in either the upper-right or lower-left VF of the screen on a background with the same color as the square array (Figure 3). Each block of trials began with a 30 s pre-adaptation period to the background’s color and luminance. Participants maintained fixation on the center of the screen during and after this pre-adaptation. 

Participants began each trial by pressing the space bar. At the start of every trial, there was a 3 s adaptation period to the pedestal contrast in the steady-pedestal paradigm (as shown in Figure 3a, top) or to the background luminance in the pulsed-pedestal paradigm (Figure 3a, bottom). Midway through the adaptation interval, a 35 ms test stimulus, in which one of the two squares in the array, either the left-most or the right-most lower square, was randomly chosen to have its luminance incremented. At the beginning of the test period, a brief tone signaled the upcoming presentation of the two squares. Participants were required to discern which square appeared brighter and indicate their choice by pressing the “left” or “right” arrow key, in a 2-alternative forced-choice task. Based on their response, the luminance of the test square in the next trial was adjusted adaptively using the QUEST procedure [53].

In both pedestal conditions, contrast-discrimination thresholds were determined by fitting a Weibull psychometric function to the test square’s contrast increment, which resulted in a 75% correct response rate. The process continued until the standard deviation of the estimated value fell below 0.05, at which point it was considered complete. Similar to our previous study [40], these thresholds typically stabilized and reached an asymptote after approximately 25 trials. 

The experiment included four blocks, formed by combining two colors (red vs. green) and two pedestal paradigms (steady vs. pulsed). In each experimental block, participants were presented with visual-field (VF) quadrants in a specific sequence, either starting with the upper-right quadrant and then moving to the lower-left, or the reverse. Additionally, the presentation order of the six pedestal contrasts within these quadrants was randomized. To ensure variability across participants, the sequence of blocks was arranged in a pseudo-random manner. Each participant conducted three replications for each condition. The threshold (Δ*C*) at each pedestal contrast was calculated as the average of the three replications for each observer, and the final threshold value was obtained by averaging across all six observers.

**Procedure (Gain-Control Computations)**. We used IBM SPSS Statistics 22.0 to obtain estimates of *R_max_*, *C_sat_*, and *δ* for each of the eight combinations of VF quadrant (lower-left and upper-right), pedestal type (pulsed and steady), and color (red and green). These estimates were selected to optimize the fit between Equation (2) and the data. Subsequently, we calculated percent contrast gain values using the formula (*R_max_*/*C_sat_*)/100. 

## 3. Results

**Figure 4:**
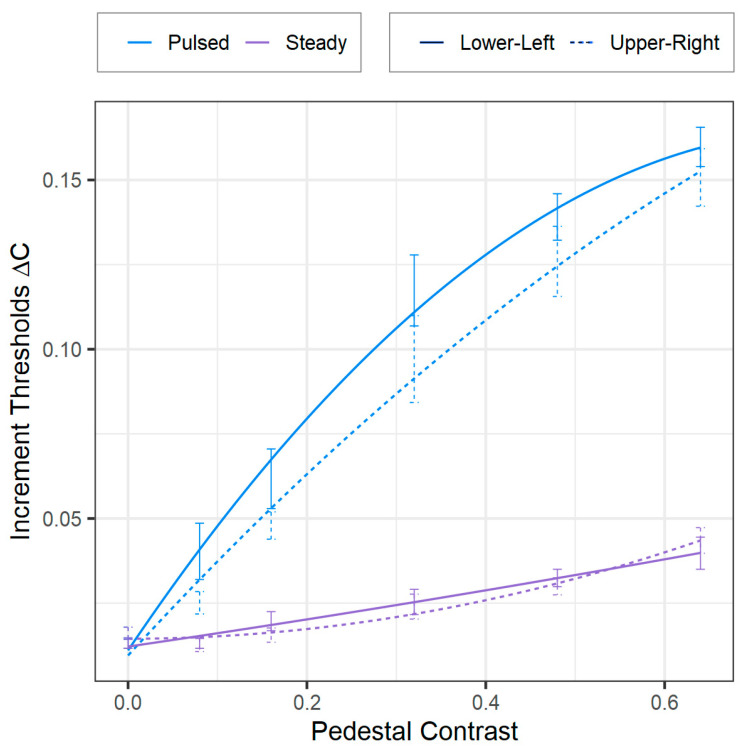
Contrast increment thresholds (Δ*C*s) as a function of pedestal contrast for pulsed-pedestal (blue) and steady-pedestal (purple) conditions in the lower-left (solid lines) and upper-right (dashed lines) VF quadrants. The curves are smoothed representations of our data (not model-fitted curves derived from Pokorny and Smith’s theoretical models). The color data are combined as they did not show statistical significance.

We carried out a four-way repeated-measures ANOVA (2 × 2 × 2 × 6) using Greenhouse–Geiser corrections for instances where Mauchly’s test showed sphericity violations. Where necessary, we employed Bonferroni-corrected post hoc analyses. This analysis, structured as two (pulsed vs. steady-pedestal paradigms) × two (background colors: red vs. green) × two (visual-field quadrants: lower-left vs. upper-right) × six (pedestal contrasts: 0.0, 0.08, 0.16, 0.32, 0.48, and 0.64), revealed significant main effects of the VF quadrant [*F*(1, 5) = 6.80, *p* = 0.048, *ƞ*^2^ = 0.58], pedestal paradigm [*F*(1, 5) = 79.76, *p* < 0.001, *ƞ*^2^ = 0.93], and contrast [*F*(5, 25) = 98.40, *p* < 0.001, *ƞ*^2^ = 0.95]. Confirming our first prediction, as shown in Figure 4, the pulsed-pedestal paradigm produced steeper Δ*C* vs. *C* functions compared to the steady-pedestal paradigm, as noted by a significant interaction between the pedestal paradigm and contrast [*F*(5, 25) = 49.09, *p* < 0.001, *ƞ*^2^ = 0.91]. (Note that Pokorny and Smith (1997) used logarithmic scales for both axes, while we used linear scales) This finding has been used previously to support the idea that the two paradigms can effectively distinguish between the P and M systems [10]. Our position that only the P system can yield such steeper Δ*C* vs. *C* functions is also strengthened, given the distinct nature of the tasks for the two paradigms. Specifically, the pulsed-pedestal paradigm requires both detection and discrimination, whereas the steady-pedestal primarily requires only discrimination.

Our second prediction was also confirmed, as there was no significant interaction between VF quadrant, contrast, and pedestal paradigm [*F*(5, 25) = 0.79, *p* = 0.57, *η*^2^ = 0.14], and no other interaction effects were observed, as can be seen in Figure 4. Significant differences in VF quadrants were seen, however, with lower Δ*C*s (indicating greater sensitivity) for the upper-right quadrant compared to the lower-left quadrant [*F*(1, 5) = 6.80, *p* = 0.048, *ƞ*^2^ = 0.58]. Our observations for the steady-pedestal paradigm diverged from Pokorny and Smith’s [10] theory. Contrary to expectations that Δ*C*s would be higher in the upper-right quadrant if the steady-pedestal paradigm were M-system biased, we observed a possible reverse trend. The lower Δ*C*s in the upper-right quadrant might suggest a P-system bias of the steady-pedestal paradigm. These results prompt a reconsideration of the paradigms, indicating a complex interaction that may favor the P-system. However, future investigations with a larger sample size are recommended. Although our sample size exceeded the number suggested by our power analysis, which was based on prior research, the results from the ANOVA might have been influenced by the relatively small sample size.

**Figure 5:**
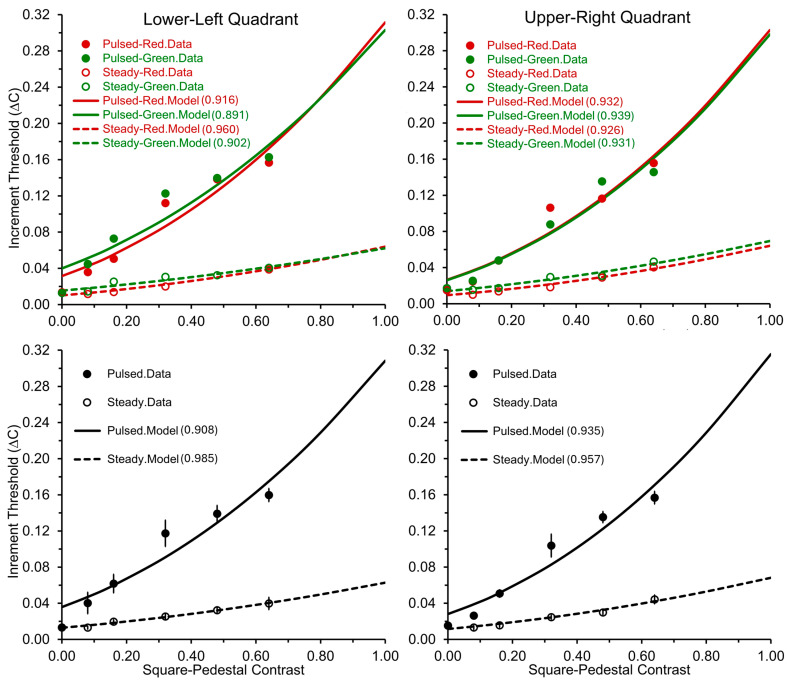
As indicated at the top of the figure, the left and right panels display contrast-increment thresholds (∆*C*s) against square-pedestal contrast for pulsed and steady pedestals in the lower-left and upper-right VF quadrants, respectively. The data for red and green stimulus colors are displayed separately in the upper panels, while the lower panels show the results averaged across the colors. Additionally, the lower panels include the standard errors of the mean (SEMs) for each data point. The curves in all panels are fitted using Equation (2) from Pokorny and Smith’s [11] models. The parenthesized values denote the R^2^ values, reflecting the best fit of Equation (2) to the respective data.

Third, in alignment with the findings of Song et al. [40] and Hugrass et al. [30], the main effect of color, as well as all of its two-way, three-way, and four-way interactions, were not significant. Figure 5 visually confirms the absence of color-related effects across the pedestal’s contrast range. However, we caution against jumping to the conclusion that the absence of noticeable differences between the effects of red and green light across both paradigms indicates a lack of suppressive effect from diffuse red light. This aspect is further explored in our discussion section, where we delve into potential reasons behind our observations in greater detail.

To assess our fourth prediction, we analyzed contrast-gain values, as depicted in Table 1, which, using Equation (2), displays the obtained best-fitting contrast-gain values. Consistent with our previous research [40], we observed that contrast-gain values were lower for the steady-pedestal paradigm compared to the pulsed-pedestal paradigm [*t*(3) = −6.95, *p* = 0.004]. This result contradicts the assumption by Pokorny and Smith [10] that the steady-pedestal paradigm reflects M-system activity.

## 4. Discussion

In our previous study [40], we discovered that Δ*C*s in both the pulsed- and steady-pedestal paradigms predominantly tracked the P-system response. Since these findings were unexpected and we used Gabor patches instead of the traditional square stimuli, we aimed to further validate the effectiveness of these paradigms using uniform-square, instead of Gabor, pedestals. In line with previous findings [30,40], color yielded no significant statistical effects. However, we suggest caution in concluding that red light does not suppress the M-system response for two reasons. First, while tentative, our data showed lower Δ*C*s in the P-biased upper-right quadrant compared to the M-biased lower-left quadrant across different conditions. This trend supports our theory over Pokorny and Smith’s [10], hinting at a complex interaction leaning towards the P-system. If the steady-pedestal paradigm is governed by the M-system, variations in response under red vs. green light in the M-biased lower-left quadrant would be anticipated. Conversely, a P-system mediation would logically result in no discernible color effect across the visual fields. This intriguing possibility calls for a deeper investigation in subsequent research endeavors.

Second, consistent with our previous study, the average contrast-gain values for the pulsed-pedestal paradigm were higher than those for the steady-pedestal paradigm. Specifically, the pulsed-pedestal paradigm yielded an average of 1.183, significantly exceeding the 0.789 average of the steady-pedestal paradigm (Table 1). This lower contrast-gain value for the steady-pedestal paradigm challenges Pokorny and Smith’s [10] argument that the steady-pedestal paradigm tracks the M-system, while the pulsed-pedestal paradigm tracks the P-system. To support their theory, the results should have indicated higher contrast-gain values for the steady-pedestal paradigm. Additionally, we conducted a comparative analysis with the data presented by Smith et al. [54], which include measurements from three observers, as depicted in Figure 2, Figure 3 and Figure 4 of their study. These observations have been previously interpreted as supportive of Pokorny and Smith’s models. Our analysis involved converting the contrast measurements from troland to Michelson contrast values, followed by the computation of contrast-gain values. The computed values were 1.115 for the pulsed-pedestal paradigm and 0.257 for the steady-pedestal paradigm, further supporting our results and challenging Pokorny and Smith’s position. For a detailed view of the converted version of Smith et al.’s [54] data, see Figure S1. 

For the reasons mentioned above, the lack of the suppressive effects of red light in prior studies [30,40] using the paradigms seems understandable if the pulsed- and steady-pedestal paradigms do not effectively differentiate between the P and M systems. Our results, using the traditional square stimuli, align with previous findings using Gabor patches [40]. While these paradigms may activate the two systems differently, our present findings suggest that the P pathway predominantly influences perceptual decisions. When assessing the P and M pathways separately in psychophysical studies, the nature of visual information and perceptual decision making critically affect the outcome. Future research should exercise caution when using these paradigms to assess the relative performances of the P and M systems, as any differences between them may be less telling than previously assumed.

## Figures and Tables

**Table 1:** Contrast gain as a function of pedestal characteristics (paradigm and VF quadrant).

Paradigm	VF Quadrant	Gain
Pulsed	Lower-left	1.297
	Upper-right	1.069
		**1.183**
Steady	Lower-left	0.838
	Upper-right	0.740
		**0.789**

Note. Numbers in bold print indicate the average of the individual contrast-gain values separately for the pulsed- and steady-pedestal conditions.

## Data Availability

The datasets generated during and/or analyzed during the current study are available from the corresponding author on reasonable request.

## Supplementary Materials

The following supporting information can be downloaded at https://www.mdpi.com/article/10.3390/vision8020028/s1, Figure S1: Smith et al.’s Data on Contrast-Increment Thresholds for Pulsed- and Steady-Pedestal Paradigms. References [10,54] are cited in the supplementary materials.
