# Supplementary material for: Further Examination of the Pulsed- and Steady-Pedestal Paradigms under Hypothetical Parvocellular- and Magnocellular-Biased Conditions
Source: Vision (Basel). 2024 Apr 30;8(2):28. doi: 10.3390/vision8020028 (PMC11130818; doi:10.3390/vision8020028)
Supplement: Supplementary file 1 [file vision-08-00028-s001.zip › vision-2874817-supplementary.pdf]

Supplementary Materials

Smith et al.'s Data on Contrast-Increment Thresholds for Pulsed- and Steady-Pedestal Paradigms

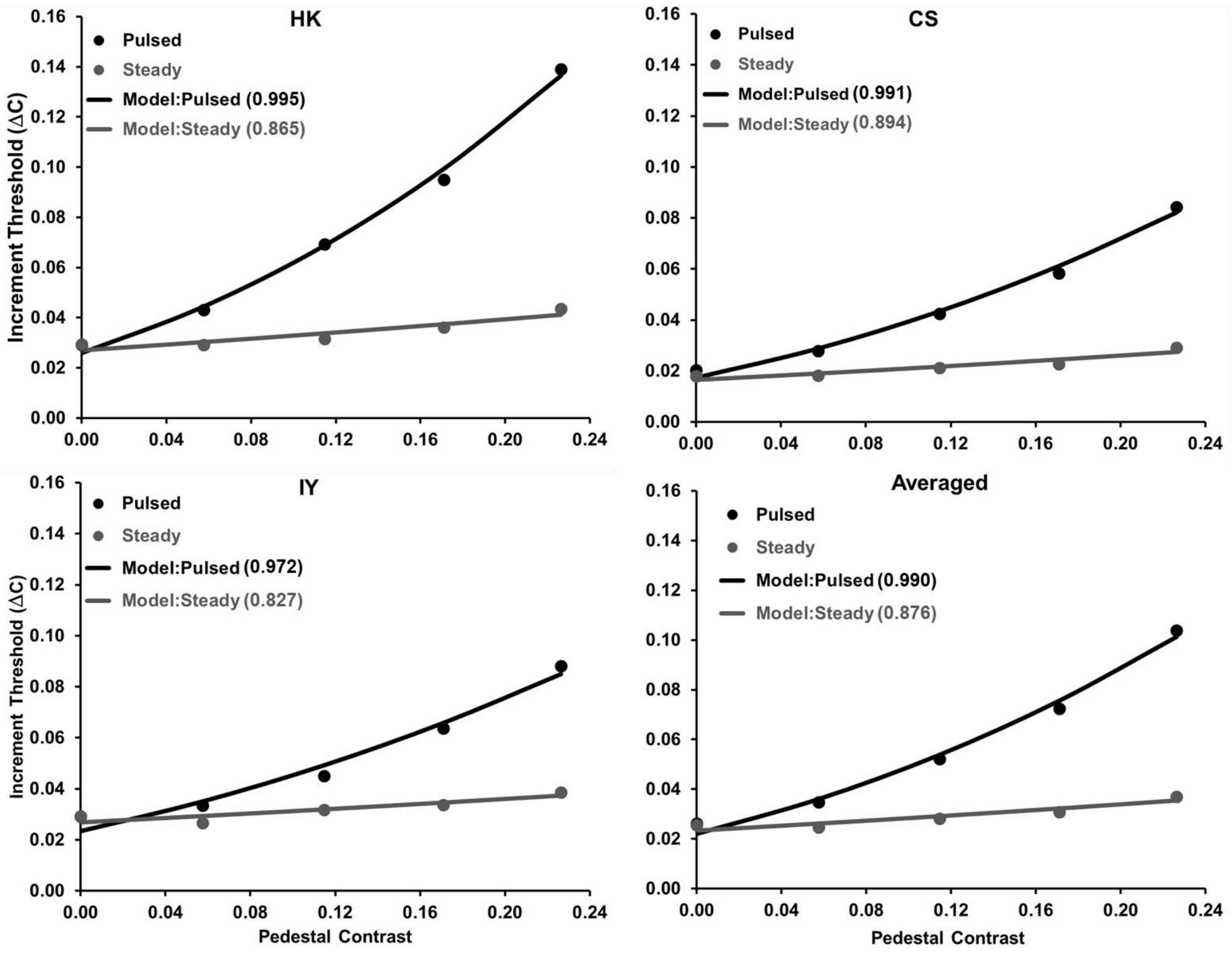

Figure S1. Contrast-increment thresholds (ΔCs) for three observers (shown in the top-left, top-right, and bottom-left panels) alongside their combined average (bottom-right panel), against pedestal contrast levels for both pulsed and steady square pedestals. These thresholds are derived from Smith et al. [54], using DataThief software (https://datathief.org/; accessed on 13 August 2022) to extract data from their Figures 2-4, each corresponding to a different observer. For all three observers in the Smith et al. study, results were first converted from ΔLuminance to Δ*C* values. For each individual observer, the Δ*C* values were then averaged over the six stimulus-array-size conditions used by Smith et al. Thereafter, the averaged results obtained for each of the three observers were in turn averaged across all three observers. The curves represent best fits obtained with Equation 2 from Pokorny and Smith [10], with standard error of the mean (SEM) shown for each data point and R^2 values in parentheses indicating the fitting accuracy of the model.
